# Biochemical enrichment and biophysical characterization of a taste receptor for L-arginine from the catfish, *Ictalurus puntatus*

**DOI:** 10.1186/1471-2202-5-25

**Published:** 2004-07-28

**Authors:** William Grosvenor, Yuri Kaulin, Andrew I Spielman, Douglas L Bayley, D Lynn Kalinoski, John H Teeter, Joseph G Brand

**Affiliations:** 1 Monell Chemical Senses Center, Philadelphia, PA 19104-3308, USA; 2 Institute of Cytology, Russian Academy of Sciences, St. Petersburg 194064, Russia; 3 New York University College of Dentistry, New York, NY 10010, USA; 4 Institute of Neurological Sciences, University of Pennsylvania, Philadelphia, PA 19104, USA; 5 Veterans Affairs Medical Center, Philadelphia, PA 19104, USA; 6 Department of Biochemistry, School of Dental Medicine, University of Pennsylvania, Philadelphia, PA 19104, USA; 7 Current Address: Department of Pathology, Anatomy & Cell Biology; Thomas Jefferson University; Philadelphia, PA 19107-6799, USA; 8 Current Address: UCSD Thornton Hospital, San Diego, CA 92037, USA

**Keywords:** Chemical senses, Taste, Signal transduction, Lectin, Ion channel, Receptor, Immunohistochemistry, Protein purification, Lipid bilayer

## Abstract

**Background:**

The channel catfish, *Ictalurus punctatus*, is invested with a high density of cutaneous taste receptors, particularly on the barbel appendages. Many of these receptors are sensitive to selected amino acids, one of these being a receptor for L-arginine (L-Arg). Previous neurophysiological and biophysical studies suggested that this taste receptor is coupled directly to a cation channel and behaves as a ligand-gated ion channel receptor (LGICR). Earlier studies demonstrated that two lectins, *Ricinus communis *agglutinin I (RCA-I) and *Phaseolus vulgaris *Erythroagglutinin (PHA-E), inhibited the binding of L-Arg to its presumed receptor sites, and that PHA-E inhibited the L-Arg-stimulated ion conductance of barbel membranes reconstituted into lipid bilayers.

**Results:**

Both PHA-E and RCA-I almost exclusively labeled an 82–84 kDa protein band of an SDS-PAGE of solubilized barbel taste epithelial membranes. Further, both rhodamine-conjugated RCA-I and polyclonal antibodies raised to the 82–84 kDa electroeluted peptides labeled the apical region of catfish taste buds. Because of the specificity shown by RCA-I, lectin affinity was chosen as the first of a three-step procedure designed to enrich the presumed LGICR for L-Arg. Purified and CHAPS-solubilized taste epithelial membrane proteins were subjected successively to (1), lectin (RCA-I) affinity; (2), gel filtration (Sephacryl S-300HR); and (3), ion exchange chromatography. All fractions from each chromatography step were evaluated for L-Arg-induced ion channel activity by reconstituting each fraction into a lipid bilayer. Active fractions demonstrated L-Arg-induced channel activity that was inhibited by D-arginine (D-Arg) with kinetics nearly identical to those reported earlier for L-Arg-stimulated ion channels of native barbel membranes reconstituted into lipid bilayers. After the final enrichment step, SDS-PAGE of the active ion channel protein fraction revealed a single band at 82–84 kDa which may be interpreted as a component of a multimeric receptor/channel complex.

**Conclusions:**

The data are consistent with the supposition that the L-Arg receptor is a LGICR. This taste receptor remains active during biochemical enrichment procedures. This is the first report of enrichment of an active LGICR from the taste system of vertebrata.

## Background

The initial event in taste transduction involves recognition of taste stimuli by plasma membrane-associated receptor proteins. These proteins are concentrated at the apical end of specialized neuro-epithelial cells (taste cells) found within multicellular end-organs known as taste buds [1,2]. The recognition binding sites for most taste stimuli face the exterior environment. The interaction of a taste stimulus with this recognition site triggers a chain of metabolic and ionic events in the taste cell, leading to alterations in membrane conductance, release of neurotransmitter, and a change in the firing rate of the afferent sensory nerve fibers with which taste cells synapse [2]. Receptor recognition is, therefore, largely responsible for maintaining the specificity of the taste transduction process.

To date, 7-transmembrane G protein coupled receptors (7TM-GPCR's) for three taste modalities have been identified by both molecular cloning and through searches of the human and mouse genome. Sweet taste stimuli appear to be recognized by at least one heterodimer (T1R2/T1R3) of the three member family of 7TM-GPCR's, the T1R's [3-7]. The taste receptors for sweetness are coupled to changes in intracellular levels of either cyclic nucleotides or polyphosphoinositols [5,8-10]. Two GPCR receptor types have been implicated in the basic taste of umami (glutamate taste). One is the heterodimer of T1R1/T1R3, of the same 7TM-GPCR family as the sweet taste receptor dimer [11]. Another GPCR umami receptor is an N-terminal truncated metabotropic-type 4 glutamate receptor (taste/mGluR4) presumably coupled to an inhibition of adenylyl cyclase [12]. A third proposed, non-GPCR umami receptor is an NMDA-type ionotropic glutamate receptor [13]. Finally, a family (~40 members) of 7TM-GPCR's recognizes many bitter taste stimuli [14,15]. These bitter taste receptors are coupled through a gustducin-containing G protein [16] to changes in intracellular levels of cyclic nucleotides and polyphosphoinositide metabolites [17-19].

While these recent discoveries have markedly improved the understanding of taste transduction, it is apparent from neurophysiological, biophysical and biochemical studies that receptors and transduction processes other than the GPCR/second messenger systems are utilized by the sense of taste [2,20]. For example, several taste transduction processes make use of ion channels as the receptor recognition step [21]. Salty taste is likely transduced by an epithelial sodium channel (ENaC), and sour taste may also make use of channels such as acid sensing ion channels (ASICs) [22] and the hyperpolarization-activated, cyclic nucleotide-gated cation channel (HCN) (reviewed by [2]). Certain stimuli, such as quinine and perhaps denatonium co-opt potassium channels to alter membrane conductance of taste receptor cells [23-25]. Finally, in a variety of species, ligand-gated ion channels have been implicated as taste receptors for a number of stimuli, including sugars in the dog [26], glutamate in mouse [13,27], nicotinamide in crayfish [28], sugars and amino acids in fleshfly [29], bitter compounds in frog [30], and apparently for amino acids in the channel catfish, *Ictalurus punctatus *[31,32]. Little is known about the structure and function of these ligand-gated ion channel receptors (LGICR) in the taste system nor the extent to which they serve as taste receptors in other species.

To evaluate the role of LGICRs in taste transduction, receptors of this class need to be identified and fully described. To date, a well characterized example of a likely LGICR class of taste receptors is found on the common channel catfish, *I. punctatus*. The catfish is an advantageous model system for studying taste transduction [33] because it possesses a large number of densely arrayed taste buds across its body surface, particularly on its barbel appendages and gill rakers [33-36], and shows high specificity and sensitivity to selected amino acids. Several taste transduction pathways for amino acids have been identified both biochemically and neurophysiologically, including those recognizing (1) L-alanine and other small neutral amino acids, (2) L-proline, and (3) L-arginine (L-Arg) [33,37-40].

Of these three receptor systems, the one tuned to the amino acid, L-Arg, appears to be of particular high specificity and affinity [38,41]. Calcium imaging studies on isolated catfish taste receptor cells suggest the presence of at least two subtypes of L-Arg-stimulated transduction pathways. In one, L-Arg induces a change in intracellular calcium that is independent of extracellular calcium activity. In the other, L-Arg induces an increase in intracellular calcium that is dependent upon extracellular calcium. This second type of L-Arg induced response is blocked by D-Arg, whereas the first type of L-Arg induced response is less sensitive to the D-isomer [42,43]. Intracellular and patch clamp studies on catfish taste cells are also consistent with there being two subtypes of responses to L-Arg [42].

The L-Arg induced increase in intracellular calcium independent of extracellular calcium and not blocked by D-Arg may utilize a mechanism such as a GPCR-polyphosphoinositol linked pathway with IP3 releasing calcium from intracellular stores. The other L-Arg-stimulated pathway, showing intracellular changes in calcium dependent upon extracellular calcium and blocked by D-Arg, is consistent with an LGICR mechanism. The LGICR mechanism was given additional credence through studies demonstrating that membranes from barbel epithelium (that contains taste buds), when reconstituted into lipid bilayers, show L-Arg-stimulated ion channel activity inhibited by D-Arg [31-33].

Using membrane homogenates from catfish barbel epithelium, biochemical binding studies revealed high affinity sites for L-Arg that were inhibited by D-Arg, L-arginine methyl ester, and to a lesser extent by L-lysine and L-α-amino-β-guanidino propionic acid [38]. Previous studies had also demonstrated that both of the lectins, *Ricinus communis *agglutinin I (RCA-I) and *Phaseolus vulgaris *Erythroagglutinin (PHA-E), inhibited the binding of L-Arg to its presumed receptor sites, and both lectins labelled identical SDS-PAGE-separated bands of a barbel membrane preparation [44]. L-Arg-stimulated ion channel activity of solubilized barbel (taste) membranes reconstituted into lipid bilayers was inhibited by both PHA-E [35] and RCA-I (Teeter & Brand, unpublished observations). In addition, PHA-E also labelled the apical membrane of selected taste bud cells and solitary chemoreceptor cells of catfish barbel [35]. When reacted against the exterior epithelium of fixed, unpermeabilized catfish barbel, polyclonal antibodies developed against these lectin reactive peptides immuno-labelled the apical membrane of a subset of barbel taste receptor cells [45].

While these prior studies are consistent with the hypothesis that one of the receptors for L-Arg is an LGICR, both the neurophysiological studies and the reconstitution experiments could not definitively test this hypothesis. Without actually isolating an LGICR for L-Arg, it remains possible that a GPCR moiety for L-Arg could conceivably be tightly coupled to a separate ion channel.

The purpose of the studies reported here was to investigate the possibility that an LGICR exists for L-Arg, by:

1. biochemically enriching this putative LGICR to near homogeneity, and

2. biophysically characterizing the L-Arg-stimulated ion channel activity of the presumed LGICR at each step of enrichment to

• demonstrate that the same LGICR is being enriched with each step, and

• demonstrate that the ion channel properties and kinetics of the enriched LGICR are similar to those of the presumed LGICR *in situ*.

This enrichment and characterization are necessary steps towards eventually cloning this putative LGICR.

The data are consistent with the interpretation that a receptor for L-Arg can be solubilized in an active state and can be enriched to a point where, upon denaturation and SDS-PAGE, material containing receptor-like activity elutes as a single band.

## Results

### Lectin specificity: lectin blots and lectin histochemistry

**Figure 1:**
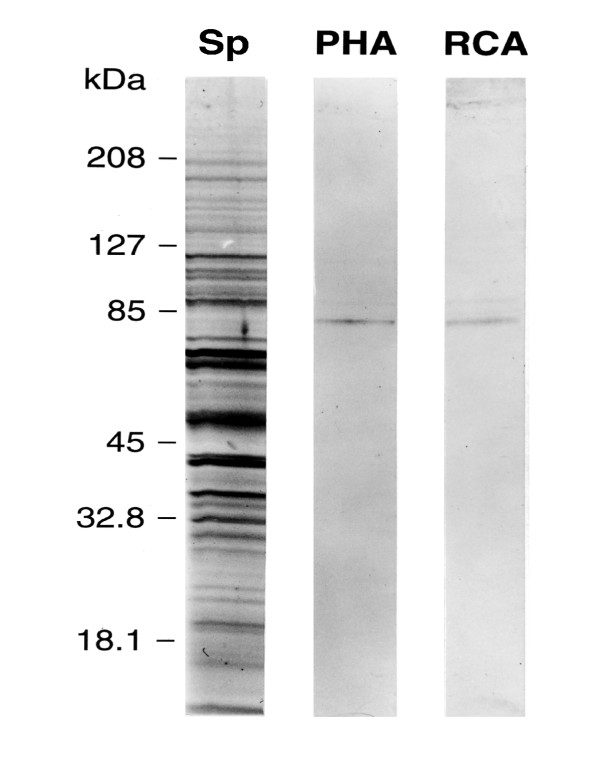
**Lectin labelling of solubilized barbel epithelial proteins **SDS-PAGE (4–20%) of 10 μg of solubilized barbel homogenate stained with silver (**Lane "Sp"**) or probed with PHA-E lectin (**Lane "PHA"**) or RCA-I lectin (**Lane "RCA"**) using lectins at 10 μg/ml with ABC detection. Both lectins label a band at 82 – 84 kDa and lightly label at least two other bands, one near 88 kDa, the other near 120 kDa.

An SDS-PAGE of a detergent-solubilized epithelial membrane fraction from barbel of *I. punctatus*, called "Sp" (defined in methods), revealed numerous proteins labelled by silver stain (Fig. 1, Lane "Sp"). Yet the lectins, PHA-E and RCA-I, both clearly labelled only one major glycoprotein band, in the range of 82 – 84 kDa (Fig. 1, Lane "PHA" and Lane "RCA"). A few other protein bands were more lightly labelled by these two lectins, including ones near 88 – 90 kDa and ~120 kDa. This recognition specificity is notable because previous work had shown that both of these lectins inhibited binding of L-Arg to a membrane suspension of barbel epithelium with respective specificity confirmed by control studies using the hapten sugar [44]. Other lectins that did not inhibit L-Arg binding labelled other glycoproteins of barbel epithelium [44].

**Figure 2:**
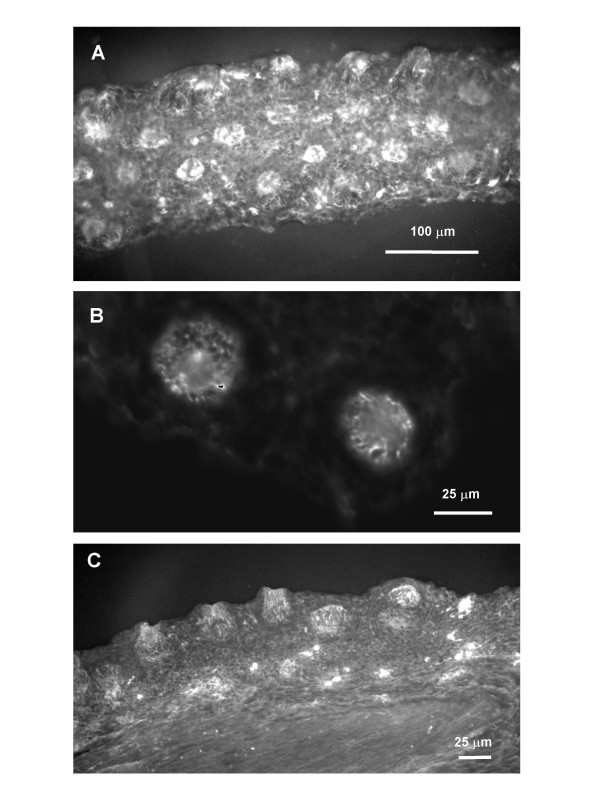
**RCA-I lectin histochemistry on barbel of catfish, *I. punctatus *(albino). **Barbels were fixed in 4% PFA, PBS, cryostat sectioned at 10 microns and histochemically probed with conjugated RCA-I at 1/200 dilution of the manufacturer's stock. **(A). **Surface labelling by RCA-I shows preferential recognition of binding sites primarily at the apical endings of taste buds. **(B). **Labelling by RCA-I of the apical region of two taste buds. (**C**). Labelling by RCA-I of horizontal section through the barbel showing reactive taste buds lining the epithelium.

Previous studies showed that the lectin, PHA-E, labelled primarily exterior-facing (presumably) glycoprotein motifs of the taste buds of catfish barbel [35]. However, no comparable labelling studies were carried out using RCA-I. Since RCA-I was used in this current study as an affinity reagent, it was important to establish the labelling specificity of RCA-I – conjugated lectin to catfish barbel taste buds. Figure 2A shows that RCA-I labels primarily the taste buds on the surface of the barbel, with Figure 2B showing labelling of two taste buds in a transverse section at higher magnification. Figure 2C demonstrates that the lectin labelled primarily the apical region of the taste bud. Some of the spotty labelling scattered among the taste bud field (Fig. 2A) may due to the RCA-I recognizing an epitope on solitary chemoreceptor cells (SCC). The SCCs are a dispersed chemoreception system found in aquatic vertebrates, and are possibly related to the taste system [35,46,47]. Apparent labelling of the catfish SCC was reported with PHA-E as well [35].

The specificity shown by these lectins in binding inhibition, ion channel conductance inhibition, lectin blots, and lectin histochemistry, all give credence to use of lectin affinity chromatography as the first step in enrichment of L-Arg-stimulated channel activity.

### Enrichment of L-Arg-stimulated ion channel activity

#### Step 1 – lectin chromatography

Since the lectins, RCA-I and PHA-E, inhibited the binding of L-Arg but not of L-alanine [44], the assumption was made that an L-arginine receptor (L-ArgR), or a fragment thereof, was among the glycoproteins labelled by these two lectins, and lectin affinity chromatography was, therefore, chosen as the first step in enrichment of this L-ArgR. Verification of the presence of active putative L-ArgR ion channel in each eluted fraction was assessed by a bilayers – incorporation assay. This assay proved to be more readily performed and provided more reproducible results than soluble binding assays that yielded high non-specific binding and required much more material.

A quantitative protein assessment of the eluted materials from the RCA-I column indicated that over 99% of the protein from fraction Sp passed through the column unbound. Only occasionally did this protein material contain minimal L-Arg-stimulated ion channel activity. In contrast, the remaining protein eluted from the affinity column by galactose and reconstituted into lipid bilayers (See below) consistently contained ion channels activated by L-Arg and inhibited by D-Arg.

**Figure 3:**
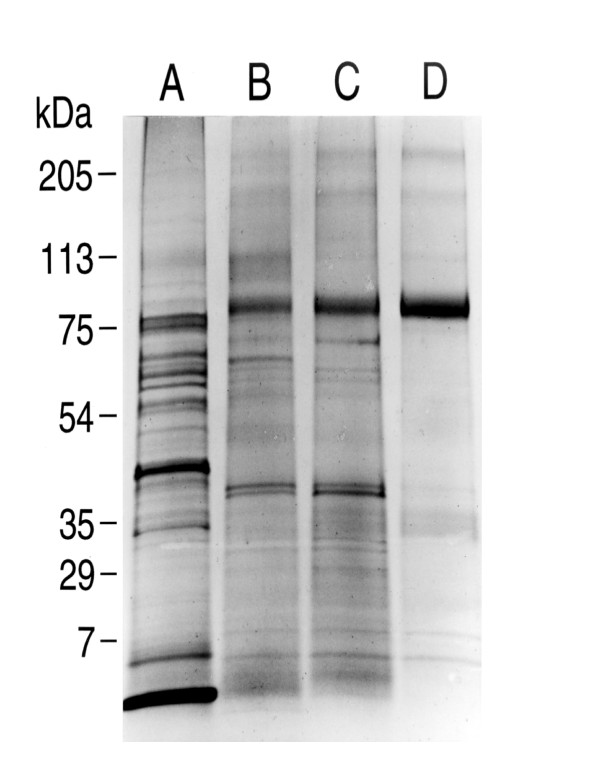
**SDS-PAGE (4–20%) of solubilized barbel protein, visualized with silver stain, before (A) and after (B, C, D) each enrichment step. **Each lane was loaded with 5 μg of protein. **Lane A: **Total protein of fraction Sp. **Lane B: **Galactose eluted protein from the RCA column containing L-Arg stimulated ion channel activity; **Lane C: **Protein of the first peak fractions of the Sephacryl gel filtration step containing L-Arg stimulated ion channel activity; and **Lane D: **Protein containing L-Arg stimulated ion channel activity from the ion exchange chromatography enrichment step.

Silver staining of SDS-PAGE separated proteins before (Fig. 3A) and after (Fig. 3B) the RCA affinity enrichment step revealed numerous proteins from total Sp (Fig. 3A) with fewer and more heavily stained bands of protein present in galactose-eluted fractions (Fig. 3B). These included primarily protein of molecular weight ~82–84 kDa, with apparently lower abundance proteins near 115–120 kDa, 60–70 kDa, and 40–45 kDa (Fig. 3B). The 82–84 kDa band matched the general position of the principal protein labelled in the lectin blots (Fig. 1). The protein eluting at 115–120 kDa in Figure 3B may correspond to the lightly labeled glycoproteins at ~120 kDa seen in the lectin blots of Figure 1. Protein of molecular weight 60 – 70 kDa and 40 – 45 kDa observed in the galactose-eluted protein fraction of Figure 3B have no apparent match in the lectin blots of Figure 1. These may, possibly, be degradation products of the other protein fractions that are labelled by the lectins or they may be proteins of low abundance, visible here due to the enrichment of protein resulting from the affinity procedure.

**Figure 4:**
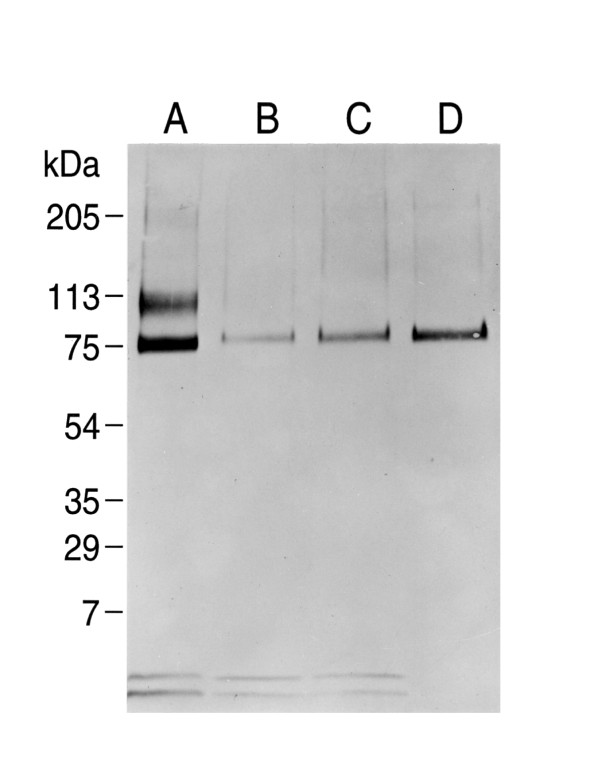
**Western blots of SDS-PAGE protein samples before (A) and after (B, C, and D) each enrichment step. **Western blots were performed using GP1. Although the identity of the samples applied to each lane was the same as illustrated in Figure 3, the amount of protein applied to each lane differed. (**A**). Fraction Sp, 5 μg, (**B**). Galactose eluted protein from the RCA column containing L-Arg stimulated ion channel activity, 1.6 μg, (**C**). Protein from the first peak fractions of the gel filtration step containing L-Arg stimulated ion channel activity, 0.2 μg, (**D**). Protein fraction containing L-Arg stimulated ion channel activity from the pH 9 fraction of the ion exchange procedure, 0.2 μg. Note that in spite of lowering protein amounts in lanes A – D, the intensity of the Western blot increases, indicative of an enrichment of the antigen protein(s) near 82–84 kDa.

On the assumption that the 82–84 kDa protein was at least a subunit of this L-ArgR, the SDS-PAGE band at 82–84 kDa was electroeluted and used to develop polyclonal antibodies from three guinea pigs (See Methods). The affinity purified and pre-treated polyclonal antibodies from two of these guinea pigs proved most specific and these were labelled, "GP1" and "GP2," respectively. In Western blots of fraction Sp (Fig 4A), both of the GP antibodies labelled a wide band between 74 and 84 kDa (containing its antigen) and occasionally a second higher molecular weight band near 110 kDa. In Western blots of RCA lectin-galactose eluted proteins, a narrower band of 82–84 kDa was labelled (Fig. 4B). Little GP labelling was seen in Western blots from SDS-PAGE of the protein not retained by the RCA lectin column, but those bands that were labelled were near 84 kDa and 110 kDa (data not shown).

The GP polyclonal antibodies were used as a confirmatory marker of the 82–84 kDa peptide during subsequent enrichment steps. Both the GP1 and GP2 antibodies labelled the 82–84 kDa protein band in Western blots of an SDS solubilized partial membrane preparation from catfish barbel and both immuno-labelled taste cells of the catfish (see ahead).

#### Step 2 – gel filtration

The CHAPS-solubilized, dialyzed, non-denatured protein eluted from the galactose wash of the lectin column was applied to a Sephacryl S-300 HR column and eluted with Tris/CHAPS. Each protein-containing peak of the elution was assayed for L-Arg-stimulated channel activity and subjected to SDS-PAGE with silver staining and Western blotting against the GP1 antibody. Only protein from the first peak, in fractions 1 and 2, (eluting at an equivalent molecular weight of > 670 kDa.) contained L-Arg-stimulated ion channel activity (see ahead). Silver staining of material in these first two fractions run on SDS-PAGE revealed a prominent band at 82–84 kDa (Fig 3C). The broad band near 110–115 kDa seen after lectin chromatography (Fig. 3B) was not seen while protein at 60–70 kDa and 40–45 kDa remained. The corresponding Western blot (Fig. 4C) demonstrated that the antigen to which GP1 was developed was still present in the active fraction.

#### Step 3 – ion exchange chromatography

As a final enrichment step eluted material from fractions 1 and 2 of the Sephacryl column were lyophilised and resuspended into Start Buffer (see methods) and loaded on a Hitrap Q anion exchange column. Both the pH 9 and pH 8 elution resulted in protein being released from the column, with the majority of protein eluting at pH9. However, only the pH 9 fraction contained L-Arg-stimulated ion channel activity when reconstituted into lipid bilayers.

A silver stain of an SDS-PAGE of the pH 9 eluent showed a deeply staining band at 82–84 kDa along with weak staining above 200 kDa and in the 35 kDa range (Fig 3D). There was also an almost complete loss of other stained bands observed in SDS-PAGE of protein from the previous enrichment steps (compare Fig. 3D with Figs. 3C and 3B). The corresponding Western blot of 0.2 μg of the pH 9 eluent showed very strong reactivity at 82–84 kDa (Fig. 4D). The SDS-PAGE of the pH 8 eluent showed a faint band at 82–84 kDa with other less intense bands at lower molecular weight. This pH 8 eluent may contain an inactive or partially denatured form of the L-ArgR.

### Immunohistochemistry of GP1 and GP2 on catfish barbel

The GP antibodies were developed against the 82–84 kDa fraction of solubilized catfish barbel membranes, since it was a band of this molecular weight that was labelled by the lectins, PHA-E and RCA-I. While it is expected that GP1 and GP2 would label an 82–84 kDa band by Western blots, the fact that the GP1 and GP2 antibodies faithfully marked each enriched fraction that exhibited L-Arg-stimulated ion channel activity (see below) suggests that they are immuno labels of the receptor. As such, their localization within the barbel may be a marker for this presumed L-ArgR.

**Figure 5:**
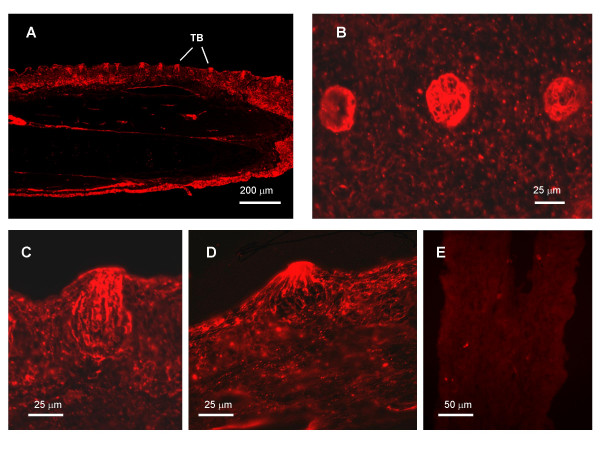
**Immunohistochemistry of catfish barbel taste buds using antibodies GP1 and GP2. **Barbels were fixed in 4% PFA, PBS, and cryostat sectioned at 10 microns. Sections were probed using indicated dilutions of antibodies GP1 and GP2. (**A). **Section through a barbel immuno-stained with GP1 at 1/8000 dilution. The lines from "TB" point to labelled taste buds. (**B**). The apical aspects of three taste buds immuno-stained with 1/12000 dilution of GP2. (**C**). Cross section through a taste bud immuno-stained with 1/16000 dilution of GP1. (**D**). A single taste bud immuno-stained with 1/8000 dilution of GP2. (E). Second antibody control showing barbel without exposure to primary antibodies.

Figure 5 shows GP1 and GP2 immuno labelling of paraformaldehyde fixed, sectioned catfish barbel. Figure 5A, a low power image of the barbel sectioned length-wise, demonstrates that it is primarily the taste buds that are labelled by GP1 (1/8000 dilution). Figure 5B shows labelling by GP2 (1/12000 dilution) of the apical area of three taste buds from a surface viewpoint. Figures 5C and 5D show taste bud labelling by GP1 (1/16000 dilution) and GP2 (1/8000 dilution), respectively of taste buds in tangential sections. Figure 5E shows a negative control where the primary antibodies were omitted. These immunohistochemical studies suggest that the antigen epitopes for GP1 and GP2 are concentrated at the apical portion of taste buds.

### Characterization of the RCA lectin-, Sephacryl S 300 gel-, and ion exchange-protein reconstituted into lipid bilayers

LGICR enrichment was followed and verified by measuring the L-Arg-stimulated conductance of lipid bilayers (equimolar mixture of DOPS:DOPE) into which the protein fractions derived from each purification step were fused. Nearly identical L-Arg-stimulated single channel activity was observed from active fractions of all three steps: the galactose-eluted protein from the RCA lectin column, the protein of the first peak, fractions 1 & 2, of the material eluted from the Sephacryl S-300 column, and the protein of the pH 9 elution from the ion exchange column. The fact that consistent and nearly identical L-Arg-stimulated activity was observed with material from each subsequent enrichment step indicates that the enrichment procedures were sufficiently benign so as to permit the retention of LGICR-type activity and, presumably, native receptor conformation.

**Figure 6:**
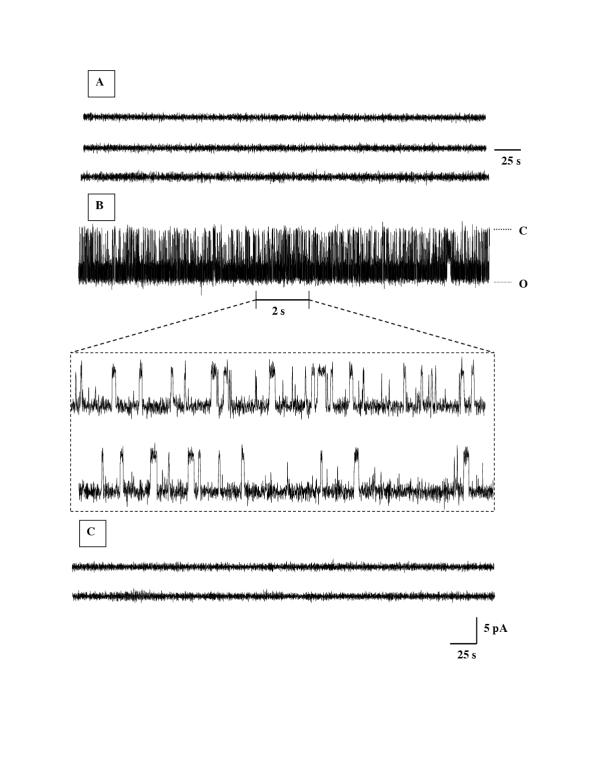
**Single channel recording of the activity of the putative L-ArgR in planar lipid bilayers. **Proteoliposomes containing protein from the first peak fractions off the Sephacryl S-300 elution were fused to planar lipid bilayers (DOPS:DOPE, 1:1) as described in Methods. **(A)**. An initial control trace was obtained after addition proteoliposomes to the membrane bathing solution, before the addition of L-Arg. The three rows are from a continuous recording. **(B)**. The addition of 10 μM L-Arg to the *cis*-side of the bilayer evoked regular periodic channel activity. A portion of this current record is shown at an expanded scale. **(C)**. After several minutes of recording, the addition of 100 μM D-Arg to the *cis*-side resulted in the cessation of activity. Transmembrane potential was -100 mV. Traces shown in all panels are continuous records of that specific condition.

General agonist/antagonist channel properties. Figure 6 illustrates single channel activities observed during the enrichment steps. Since active material from each of the three steps yielded almost identical channel properties (See Table 1), only that activity seen with fractions 1 and 2 (combined) off of the Sephacryl S-300 column is shown here. The data of Figure 6 are from the same experiment.

In lipid bilayers into which active material had been fused, but in the absence of added L-Arg, no spontaneous channel activity was observed (Fig. 6*top panel*). Addition of 10 μM L-Arg to the *cis *side buffer solution induced appearance of ion channels (Fig. 6*middle panel*). L-Arg-stimulated channel activity in positive fractions from all columns was readily blocked by the addition of 100 μM D-Arg to the same side of the chamber wherein L-Arg had been added (Fig. 6*bottom panel*). (In control experiments with bilayers into which no protein was incorporated, neither L-Arg nor D-Arg alone (in a range 10 – 1000 μM) induced channel activity.)

While 10 μM L-Arg was generally used in the screening and assay procedures, we estimate that the threshold for L-Arg-induced channel activity of the solubilized putative L-ArgR is about 1 μM L-Arg.

In addition to inhibition by D-Arg, the lectins, RCA-I and PHA-E (not shown here, but see [35]), also inhibited the L-Arg-induced ion channel activity. In contrast, none of the antibodies developed to the denatured 82–84 kDa proteins inhibited L-Arg stimulated ion channel activity. Neither L-alanine nor L-proline (up to 200 μM) activated the ion channels stimulated by L-Arg.

#### Amplitude histogram

**Figure 7:**
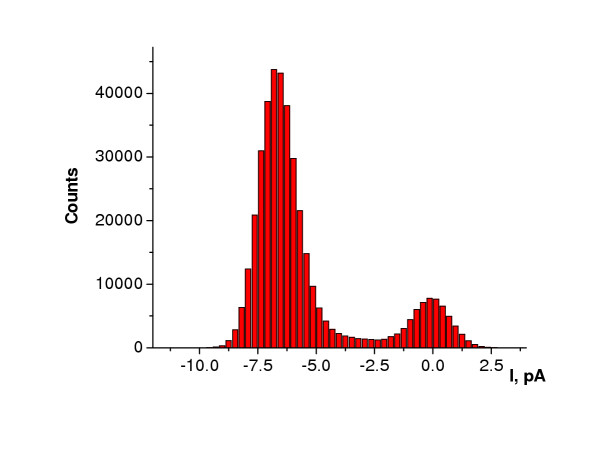
**Amplitude histogram of current fluctuations of the putative L-ArgR in lipid bilayers. **Current fluctuations were calculated from studies similar to those presented in Figure 6, part **B**, using the same protein fraction, measured at -100 mV transmembrane potential. The histogram was fit by a Gaussian distribution and mean current values were obtained from the center of the distribution. Bilayer was DOPS:DOPE, 1:1.

Figure 7 illustrates an all points amplitude histogram of L-Arg activated channels from material contained in fractions 1 & 2 from the Sephacryl column elution step measured at a fixed transmembrane potential of -100 mV. These data imply that there is one major peak of current amplitude (with some fluctuation) giving a unitary current of -6.6 pA. Similar unitary currents were measured for material from both the lectin column and the ion exchange procedure.

#### Cation/anion selectivity & current/voltage relationship

**Figure 8:**
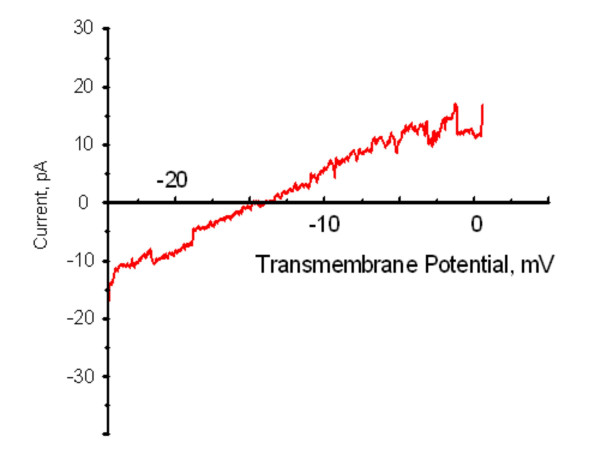
**Cation-anion selectivity of L-ArgR channels. **The figure shows current elicited by voltage ramps across a lipid bilayer (DOPS:DOPE, 1:1) containing several channels from the Sephacryl S-300 step, in the presence of a 4-fold gradient of NaCl across the membrane (*cis *and *trans *chambers contained 100 and 25 mM NaCl respectively). The reversal potential is -14 mV, corresponding to weak cation selectivity (P_Na_/P_Cl _= 2.2).

The cation-anion selectivity of L-Arg activated channels found in material contained in fractions 1 & 2 off the Sephacryl column was determined for Na^+ ^and Cl^-^. A potential of zero current (reversal potential) was measured after formation of a 4-fold transmembrane concentration gradient of electrolyte (100 mM NaCl at *cis *side and 25 mM NaCl at *trans *side) across the bilayer containing a few ion channels. The average reversal potential (n = 5 bilayers, ± S.D.) elicited by voltage ramps (see Fig. 8) was -14 ± 3 mV. This value corresponds to weak cation selectivity (P_Na_/P_Cl _= 2.2).

**Figure 9:**
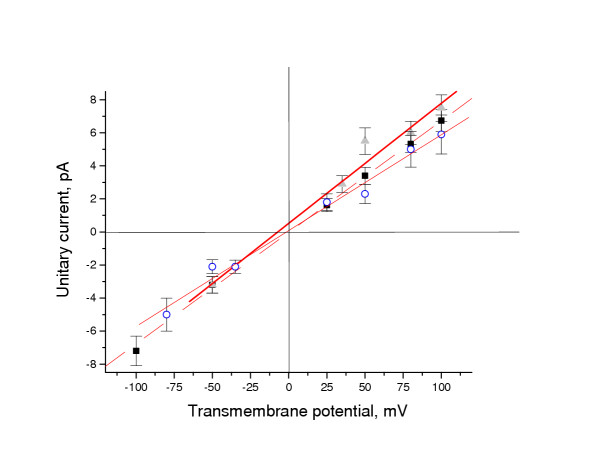
**The unitary current/voltage (I-V) relationship of channels formed by protein of each enrichment step. **The I-V relationships were obtained using L-Arg – active protein from the RCA-I lectin column (o) from the first peak fractions off the Sephacryl S-300 HR column (■) and from the pH 9 elution of the ion-exchange column (▲). Measurements were made under symmetrical conditions of 100 mM NaCl, 1 mM CaCl_2 _and 5 mM MOPS (pH = 7.2). Data points indicate the Mean ± S.D. The data sets are well fit by a linear regression (*r *= 0.99 solid and dotted lines) with slopes of 58, 67, and 73 pS respectively. Bilayer was DOPS:DOPE, 1:1.

The current-voltage relationships for L-Arg activated channels formed by active protein fractions from all three enrichment steps are illustrated in Figure 9. The data are well fit by a linear regression (r = 0.99) with slopes of 58, 67, and 73 pS for channels formed by protein from the RCA-I lectin column (open circles), protein from Sephacryl S-300 HR column (solid boxes) and protein from the pH 9 fraction of the ion-exchange column (triangles), respectively. From these current-voltage relationships it follows that the conductance of the channels from protein at any stage in the enrichment process are nearly identical, and that L-Arg activated ion channels are essentially potential-independent.

#### Comparison with biophysical properties of native channels

The electrophysiological properties of ion channels formed by the protein fractions throughout the enrichment scheme described here closely resemble those measured for the native channels [31] (see Table I). Both native channels and channels formed by proteins after these enrichment steps

1. are activated by L-Arg and inhibited by D-Arg over the same concentration ranges;

2. display nearly the same unitary conductance (The membrane-associated channels show two conductance states, one at 40 – 60 pS, the other at 75 – 100 pS (Table I and [31]));

3. are cation selective; and

4. are potential independent.

## Discussion

Ligand-gated ion channel receptors (LGICR) may be used for selected stimuli of several taste modalities. In spite of their likely role in taste, little is known about these LGICRs [21]. The best characterised apparent taste LGICRs that recognise non-ionic stimuli are those of the catfish, *I. punctatus*. This animal possesses apparent LGICRs of low affinity for L-proline and of high affinity for L-Arg [31,33].

The observation that L-Arg acts as a stimulus for the taste system of the channel catfish was first reported by Caprio [48]. Subsequent neurophysiological and biochemical binding studies demonstrated that, unlike most other vertebrate taste receptors, the catfish taste receptor(s) for L-Arg is of both high specificity and high sensitivity [32,38,41]. Contemporaneous neurophysiological cross-adaptation and single unit studies indicated that L-Arg stimulates unique sites independent of those for other amino acids such as L-alanine or L-proline [49,50,40].

The receptor sites for L-Arg have narrow structural requirements, with only a few structural analogs of L-Arg acting as cross-adapting stimuli [41]. The receptor binding studies found a high affinity site for L-Arg, with Kd of 20–50 nM, and demonstrated inhibition of L-Arg binding by D-Arg, L-arginine methyl ester, and to a lesser extent by L-lysine and L-α-amino-β-guanidino propionic acid [38]. Other amino acids were without effect at reasonable levels. Interestingly, L-Arg and D-Arg are non-reciprocal cross adaptors, where neural adaptation to D-Arg eliminates responses to L-Arg, while adaptation to L-Arg still leaves some response to D-Arg [40]. This non-reciprocal cross-adaptation predicts the presence of a receptor site for D-Arg and suggests that any receptor for L-Arg – be it a GPCR or an LGICR – should be sensitive to D-Arg. The fact that no responses to D-Arg were ever observed in the bilayer experiments suggests that the major receptor for D-Arg is a GPCR. Consistent with these receptor specificities is the behavioural observation that at micromolar levels, L-Arg induces oropharyngeal motor behavior in *I. punctatus *[33,51,52] with D-arginine acting as a partial antagonist of this behavior [33].

More recently, whole cell patch clamp and calcium imaging of isolated catfish taste receptor cells, along with earlier *in situ *intracellular electrophysiological recordings, indicated that the majority of L-Arg-induced depolarizations are generated by inward currents [42,43]. As predicted by neurophysiological cross-adaptation studies [40] and consistent with biochemical binding experiments [38] the increases in intracellular Ca^2+ ^activity observed in taste cells stimulated by L-Arg could be blocked by D-Arg [43].

Studies in our laboratory demonstrated that plasma membrane vesicles from barbel epithelium incorporated into lipid bilayers displayed L-Arg (μM)-stimulated ion channel activity that was inhibited by D-Arg [31,32,35]. No channel activity was observed toward L-alanine, but another, apparently less abundant, channel was stimulated by mM levels of L-proline, with the L-proline response being inhibited by D-proline. The L-Arg-stimulated responses were not inhibited by D-proline, nor were the L-proline responses inhibited by D-Arg [31]. The L-Arg-stimulated channels were found to be 50–80 pS in size, cation selective, but of low ion specificity. In contrast to the taste system, the olfactory system of the catfish transduces the stimulus, L-Arg and some other basic amino acids apparently through a GPCR [53] as does the olfactory system of goldfish [54]. In addition, L-Arg is an appetitive stimulus for the leech, Hirudo medicinalis, where the transduction process for L-Arg can be influenced by bitter stimuli, suggesting an integration at the receptor cell level [55]

Preliminary reports on the localization of an L-ArgR showed that the antibodies, GP1, and the lectin, PHA-E, when incubated with intact, unfixed barbels, labelled exterior-facing epitopes on catfish barbel taste buds [35,45] and SCCs scattered in the epithelium among taste buds [35]. Immunoelectron microscopy using GP1 revealed labelling primarily on those cells of the taste bud containing large microvilli [35]. Because these data were of surface labelling only, the labelling specificity towards other areas of the taste bud and the barbel epidermis for GP1 and the lectins was not known. This current report demonstrates primarily apical labelling of taste buds by both conjugated RCA-I, GP1 and GP2 (Figs 2 and 5). The scattered punctuate labelling seen with both RCA-I and the GP antibodies may represent epitope recognition on solitary chemoreceptor cells (particularly within the epidermis), the apical processes of which were previously shown to label with PHA-E [35]. As the secondary antibody controls suggest (Fig. 5E) very little of this punctuate labelling can be attributed to a second antibody effect.

Considering collectively the neurophysiological, biochemical, behavioural, biophysical and localization data, a putative receptor for L-Arg emerges as one of high structural specificity, with D-Arg acting as an antagonist, one of high sensitivity, and one expressed in the apical membrane of a specific sub-class of taste receptor cells. Yet, because the previous biophysical studies were carried out with intact cells, epithelial homogenates or reconstituted membrane vesicles, the data are insufficient to permit a distinction between the L-ArgR as a single LGICR macromolecular complex and the receptor as two separate entities, a recognition molecule coupled to ion channel activity. To help make this distinction, solubilization and enrichment of the receptor were required. We assumed that if receptor activity survived solubilization and increasing enrichment, and if this receptor appeared to purify as a unitary entity by SDS-PAGE, then it is likely that the major receptor for L-Arg is indeed a LGICR.

This enrichment procedure was also a necessary first step in cloning the receptor, since partial amino acid sequences may be obtained from the purified product.

### Enrichment of the putative L-ArgR

The initial enrichment step of lectin affinity was predicated upon the observation that the lectins, RCA-I and PHA-E, inhibited the specific binding of L-Arg to its presumed receptor sites [44], and that PHA-E and RCA-I inhibited L-Arg-stimulated conductance activity of catfish barbel (taste) membranes reconstituted into lipid bilayers [35]. In addition, both lectins labelled only a few protein bands of an SDS-PAGE of barbel Sp, and only one major band, that near 82–84 kDa, was common between the two (Fig. 1). Both conjugated RCA-I and PHA-E labelled cells within the taste buds (Fig. 2 and [35]).

Lectin affinity chromatography employing agarose bound RCA-I was therefore used to achieve an initial partial enrichment of the putative L-ArgR. RCA-I was chosen for lectin affinity because in our hands it was more stable and easier to work with than the agarose bound PHA-E. In pilot studies, lectin affinity chromatography with PHA-E led to results similar to those obtained with RCA-I.

The two additional enrichment procedures using Sephacryl-S300 and Hitrap Q ion exchange were chosen to further enrich the putative L-ArgR because both could be carried out using solubilization buffers that were less likely to destroy the activity of the receptor and both are standard biochemical purification techniques. Each chromatography step that retained L-Arg-stimulated ion channel activity resulted in increasingly concentrated and increasingly purified protein of molecular weight near 82–84 kDa. This enrichment is readily seen in SDS-PAGE of Figure 3, where the protein profile changes dramatically over the course of each chromatography step. The Western blots of material from each step (Figs. 4A,4B,4C,4D) suggest a substantial enrichment of the 82–84 kDa protein.

Throughout enrichment, the 82–84 kDa band was consistently recognized by the GP1 and GP2 antibodies in Western blots (Fig. 4). These blots suggest that the same entity(ies) labelled by the lectins was retained and enriched through the course of each chromatography step. The Western blots also speak to the specificity of the antibodies, GP1 and GP2, in that both antibodies labelled almost exclusively the 82–84 kDa band. The subsequent observation that GP1 and GP2 recognized epitopes at the apical region of the taste bud (Fig. 5) indicates that at least a portion of the proteins in the 82–84 kDa region are taste cell-related, membrane associated and, given the biophysical characteristics, likely receptors.

While the 82–84 kDa protein(s) may be a major constituent of the active ion channel complex, the actual molecular weight of the active receptor, and therefore some estimate of its quarternary composition, was difficult to determine. Attempts at running native gels led to inconsistent findings. Of the procedures used for enrichment, the Sephacryl column was the one that could, theoretically, at least, give an estimate of the size of the complex. Using this column, all of the L-Arg-stimulated ion channel activity was located within the initial eluted peak (fractions 1 & 2). Calibration of the column suggested a molecular weight of > 640 kDa for eluted material at this initial peak. However, this high apparent molecular weight may not represent the actual weight of the unitary LGICR, since, like many other LGICRs, the L-ArgR may form clusters [31,56-58]. Theoretically, use of Sephacryl 400HR should be able to resolve such high molecular weights. However, when Sephacryl 400HR was used, ion channel activity was spread across many eluted fractions, making estimates of unitary molecular weight impossible.

After these enrichment procedures, the L-Arg-stimulated ion channel activity was retained and the 82–84 kDa protein fraction was greatly purified. The correlation of these two observations suggests that protein in the 82–84 kDa range is at least part of the L-ArgR.

### Biophysical characteristics of the putative L-ArgR

The biophysical characteristics of the L-Arg-stimulated channels reconstituted from each step in the enrichment scheme remained nearly unchanged from those described for the channel observed in native membrane fragments [31,32]**:**

1. both channels were activated by the same range of concentration of L-Arg and blocked by the same concentration range of D-Arg (Fig. 6);

2. neither were activated by L-alanine nor by L-proline;

3. both displayed similar amplitude histograms (Fig. 7 and [31]);

4. both had similar unitary conductance (see Table 1), with the enriched channel displaying unitary conductance of 73 +/- 7 pS (Fig. 7), and the channel in situ displaying two conductance ranges, 40 – 60 and 75 – 100 pS. This difference is likely due to the difference in buffers, where the bilayer studies were performed in NaCl/CaCl2/MOPS, while the reconstituted membrane in situ studies were performed with a complex ringer buffer (Table 1, Fig. 8);

5. both channel preparations when stimulated by L-Arg exhibit linear current voltage relationship (Fig. 9).

The results of this study demonstrate that the isolated channel protein shows recognition-specificity for L-Arg and acts as a non-specific ion channel upon binding L-Arg, properties consistent with the *in situ *activity of the putative L-ArgR and consistent with expected taste receptor criteria.

## Conclusions

Collectively, the data presented here suggest that one major taste receptor for L-Arg in the catfish, *I. punctatus, *is a ligand-gated ion channel receptor. The active receptor was biochemically enriched from taste bud-containing epithelium and biophysically validated. Immunohistochemical studies using an antibody raised against peptides labelled by lectins that inhibited the binding of L-Arg to a likely receptor revealed specific labelling at the apical region of the taste bud. Analogous with other LGICRs, taste receptor cell depolarization to L-Arg is suggested through L-Arg binding to a receptor site that is associated with and activates an ion channel of low ion selectivity. In the animal, activation of this channel by L-Arg or other select agonists will open the channel and allow influx of Na^+ ^and Ca^2+^, present in the mucus covering the taste epithelium [32], into the receptor cell. Alternatively, given the relatively low cation/anion ratio, at least part of this charge could be carried by efflux of Cl^-^. This flow of charge will result in cellular depolarization, release of neurotransmitter to the innervating sensory nerve, and transmission of the taste signal to the central nervous system. This enrichment procedure can be used to generate sufficient material for obtaining partial peptide sequences necessary for eventual cloning of this LGICR.

## Methods

### Animals

Use of the channel catfish for these studies was approved by the Institutional Animal Care and Use Committee of the Monell Chemical Senses Center. Channel catfish, *Ictalurus punctatus*, purchased from local suppliers were usually euthanized on the day of delivery, but if not, were held less than 4 days in 250 gallon aquaria under dim light and fed commercial catfish chow.

### Chemicals

All electrolytes, buffers and other chemicals were reagent grade from Sigma (St. Louis, MO). Water was deionized and further purified through a Milli-Q Plus PF system (Bedford, MA). 3-[(3-Cholamidopropyl)-dimethylammonio]-1-propanesulfonate (98%) (CHAPS), polyoxyethylenesorbitan monolaurate (TWEEN 20), phenylmethanesulfonyl fluoride (PMSF) and pepstatin A were purchased from Sigma. n-Octyl-β-D-gluco-pyranoside (n-octylglucoside) was purchased from Calbiochem (LaJolla, CA). The absolute enantiomer, L-arginine HCl, was purchased from Sigma, and the absolute enantiomer, D-arginine HCl, was a gift of the Ajinomoto Co., Tokyo, Japan. Sephacryl S-300 HR, High Range Gel Filtration Calibration Kits, and 1.0 ml Hitrap Q columns were purchased from Pharmacia Biotech (Piscataway, NJ). Agarose-bound lectins, *Ricinus communis *agglutinin I (RCA-I), *Phaseolus vulgaris *Erythroagglutinin (PHA-E), in their biotinylated forms, the ABC kits, and rhodamine-conjugated RCA-I were purchased from Vector Lab (Burlingame, CA). The second antibody, Cy3-conjugated goat anti-guinea pig IgG, was obtained from Jackson ImmunoResearch Labs. (West Grove, PA). The 4 CN Membrane Peroxidase Substrate System was purchased from Kirkegaard & Perry Laboratories (Gaithersburg, MD). Gels and ampholytes were purchased from BioRad (Hercules, CA). Protein was quantitated using a BioRad DC Protein Assay. Synthetic 1,2-dioleoyl-*sn*-glycero-3-phosphoserine (DOPS), 1,2-dioleoyl-*sn*-glycero-3-phosphoethanolamine (DOPE) and 1,2-dioleoyl-*sn*-glycero-3-phosphocholine (DOPC) were purchased from Avanti Polar Lipids, Inc. (Pelham, AL).

### Tissue homogenate preparation and solubilization

Maxillary and mandibular barbels from approximately 50 euthanized channel catfish, ~25–40 cm long, were removed and placed in a beaker containing 100 ml of 50 mM Tris-HCl buffer (pH = 7.8), 100 mM NaCl, 1 mM EDTA ("TRIS Wash"). After one exchange of buffer, barbels were removed to a 50 ml polypropylene screw cap tube filled with TRIS Wash buffer and stored at -80°C until used.

The preparation of a solubilized, plasma membrane-enriched fraction from catfish barbels was adapted from Kalinoski [59]. A typical homogenate/solubilization run used barbel tissue from 150 fish, prepared in aliquots of 50 fish at a time. The entire procedure was carried out at 4°C. The epithelium of thawed barbels from 50 fish at a time in 75 ml of TRIS Wash was stripped from the supporting pseudo-cartilage with two 10 second bursts (with a 10 second interval) from a hand-held Toastmaster Hand Blender, Model 1738 (Boonville, MO). The suspension was allowed to settle, and the supernatant decanted into a 600 ml beaker through two layers of USP Type VII gauze (Kendall Co., Boston, MA). Fifty ml of TRIS Wash (4°C) was added to the remaining settled barbels, and the suspension subjected to a third 10 second burst from the Blender. This entire second suspension was rapidly poured over the gauze layers into the same beaker. A second and third tube of barbels from 50 fish were treated identically, and the filtrate from all three combined, and the volume brought to 470 ml. This homogenate, divided into two aliquots, was centrifuged at 4000 × g for 15 min. The supernatants were recovered and centrifuged at 21,500 × g for 45 min. The pellets were retained.

To solubilize the pellets from the 21,500 × g spin each pellet was recovered by two, 1 ml rinses of 50 mM TrisHCl (pH 7.8), 50 mM NaCl ("Low Osmolar Buffer") (total, 4 ml) and transferred to a 15 ml Teflon/glass homogenizer. Twenty milligrams of CHAPS and 40 μl of protease inhibitor-mix (0.275 mM pepstatin A, 57.5 mM PMSF, in ethanol) were added to the ~4 ml suspension in the homogenizer. The suspension was homogenized by ten slow strokes of the Teflon pestle using a rotating motor drive at moderate speed. The homogenate was transferred to a 15 ml capped tube, diluted to 10 ml with Low Osmolar Buffer, and placed on a Clay Adams Nutator rocker/shaker overnight at 4°C to solubilize the proteins. After the overnight agitation, the suspension was centrifuged for 1 h at 100,000 × g. The supernatant was recovered and is referred to as "Sp." This Sp was then used in the subsequent lectin affinity chromatography step.

### Lectin affinity chromatography

All steps in the lectin affinity procedure were performed at 4°C. Agarose-bound RCA I affinity resin was pre-washed by adding 8 ml of gel slurry to a 125 ml conical glass tube and bringing the final volume to 14 ml with TRIS Wash. The tube was inverted several times, then centrifuged at 500 × g for 1 min. The supernatant was discarded and the wash step repeated three additional times with TRIS Wash buffer, then two more times with Low Osmolar buffer. The resulting agarose-bound RCA I gel, with a bed volume of 4 ml, was used for affinity chromatography.

The 4 ml agarose-RCA gel in the 15 ml tube was combined with ~7 ml of Sp, and the mixture equilibrated on the Nutator tube rocker for 30 min. The mixture was poured into a 1 cm × 10 cm column and the effluent collected as 1 ml fractions at a flow rate of 6.0 ml/h. Effluent from the column was monitored by absorbance at 230 nm and/or 280 nm.

To remove unbound protein, the column was washed with a sufficient volume (~12 ml) of Low Osmolar Buffer until no detectable protein eluted from the column. Proteins bound to the RCA resin were then eluted from the column with 10 ml of 50 mM Tris-HCl (pH = 7.8), 200 mM NaCl, 1 mM EDTA, 20 mM D-galactose. Protein from both the Low Osmolar elution step and the D-galactose elution step was reconstituted into lipid bilayers and assayed for L-Arg-stimulated ion channel activity (See ahead).

The galactose-eluted protein fractions showing L-Arg-stimulated ion channel activity were pooled and dialyzed over night against 2000 ml Milli-Q water containing 0.05% CHAPS. Dialyzed samples were lyophilized and stored at -80°C.

### Gel filtration chromatography

All steps in the gel filtration procedure were performed at 4°C. Lyophilized proteins from the galactose elution of the lectin column were prepared for further enrichment using size exclusion chromatography by dissolution in 300 μl of TRIS Wash with the addition of 0.2% CHAPS and 10% sucrose. This preparation was loaded onto a Sephacryl S-300 HR column (1 cm × 40 cm). Fractions of 0.4 ml were eluted from the column with a buffer composed of TRIS Wash plus 0.2% CHAPS. The column was run at 2.4 ml/hr, and the effluent absorbance monitored at 230 nm and 280 nm. Eluted fractions containing measurable protein were evaluated for L-Arg-stimulated ion channel activity by incorporation into a lipid bilayer (See ahead). In addition, SDS-PAGE (See ahead) was performed on each eluted fraction. Those fractions exhibiting L-Arg-stimulated ion channel activity were dialyzed overnight against 2000 ml of Milli-Q water containing 0.05% CHAPS, lyophilized and stored at -80°C.

The column was calibrated with a Gel Filtration Calibration Kit with protein standards from 158 to 669 kDa.

### Ion exchange chromatography

All steps in the ion exchange procedure were performed at 4°C. Further enrichment of the fractions exhibiting L-Arg-stimulated ion activity was achieved using an anion exchange column (Hitrap Q). The column was prepared by washing with 5.0 ml of Start Buffer (25 mM Tris-HCl, pH = 9.0), followed by 2.0 ml of Regeneration Buffer (Start Buffer plus 1.0 M NaCl) and then 5.0 ml of Start Buffer. Lyophilized proteins of the active fraction from the gel filtration column were dissolved in 300 μl of Start Buffer and applied to the column. Prior to elution, the loaded column was washed with 2.0 ml Start Buffer. Elution was at 12 ml/h in 400 μl fractions. First, 1.0 ml of First Elution Buffer (25 mM Tris-HCl, pH = 9.0, 500 mM NaCl) was applied to the column followed by (second), 2.0 ml of Start Buffer (no NaCl), followed by (third) 4.0 ml of Second Elution Buffer (25 mM Tris-HCl, pH = 8.0, 500 mM NaCl). Effluent from the column was monitored with in line UV (230 and 280 nm) and conductivity detectors. Each fraction was assayed for L-Arg-stimulated activity (See ahead.). The active fractions were pooled, dialyzed overnight against 2000 ml of Milli-Q water containing 0.05% CHAPS, lyophilized, and stored at -80°C.

### Development of polyclonal antibodies

The procedure for development of antibodies against the catfish barbel peptides labelled by the lectins, RCA-I and PHAE has been described previously [45]. Briefly, electroeluted material from that area of a gel congruent with an identical gel labelled by the lectins was injected into three female guinea pigs using a schedule and procedure previously found to raise high titer polyclonal antibodies [60]. Antisera were aliquoted into 500 μl lots in 1.5 ml Eppendorf tubes and kept frozen at -80°C until used. Antisera from animal #1 (GP1) and animal &2 (GP2) were found to be the most specific in that they reacted primarily with their antigen within the 82–84 kDa band in Western blots of SDS-PAGE of catfish barbel membranes.

The IgG fraction of GP1 and GP2 antisera was purified using an E-Z-SEP antibody purification kit (Pharmacia). To reduce non-specific binding in the immunohistochemical studies and Western blots, the GP1 and GP2 antibodies were incubated with powder derived from an acetone precipitation of a catfish brain homogenate. One ml of E-Z-SEP-purified antibody was incubated with 10 mg of powder for 40 min at 4°C. The powder was removed by centrifugation and the procedure was repeated once with fresh powder. The resulting pretreated antibodies are called simply, "GP1" and "GP2."

### Gel electrophoresis, lectin blots and Western blots

Tris-glycine gels (4–20%, Bio-Rad) were used for SDS-PAGE. Protein of fractions before any chromatography (i.e., Sp), as well as those from each chromatography step, were denatured by mixing 10 μl from each fraction, 1:1, with sample buffer containing 125 mM Tris-HCl (pH = 8.0), 20% glycerol, 4% SDS, 4% β-mercaptoethanol and 50 μg/ml bromophenol blue, and placing the mix in a boiling water bath for 5 min. Gels were run at a constant 20–25 mA for about 1 h, using prestained broad range molecular weight markers (Bio-Rad) in 1 or more lanes. Proteins were stained with Bio-Rad Silver Stain Plus kit.

For lectin blots, proteins were electrophoretically transferred to nitrocellulose sheets (Bio-Rad Mini Trans-Blot, Hercules, CA). The sheets were incubated in a blocking solution (2% gelatine, phosphate buffered saline [(PBS) (150 mM NaCl, 100 mM sodium phosphate, pH = 7.4)] and 0.05% Tween 20) for 2 h at room temperature, and then incubated with biotinylated RCA-I or PHA-E (10 μg/ml) overnight at 4°C. Following exposure to biotinylated lectin, nitrocellulose sheets were washed extensively with blocking solution. Lectin-bound protein bands were visualized using a Vectastain peroxidase ABC kit with a 4 CN Membrane Peroxidase Substrate system. Development was stopped after one hour using 5% glacial acetic acid.

For Western blots, proteins were transferred to nitrocellulose and incubated in blocking solution (PBS, pH = 7.4, 5% non-fat dry milk, 1% goat serum and 0.05% TWEEN 20) for 2 h at ambient temperature with constant slow rocking (Nutator). The nitrocellulose was incubated overnight at 4°C (rocking) with primary antibody (GP1, 1/500) in blocking solution. The nitrocellulose was washed with blocking solution and incubated with biotinylated secondary antibody (1:250) for 1 h with slow rocking. Bands were visualized as above.

### Lectin histochemistry and immunohistochemistry

Rhodamine-conjugated RCA-I (Vector Labs) was used to estimate the specificity of lectin interaction with glycoproteins of catfish barbel. To assess the localization of the antigen contained in the 82–84 kDa band from the SDS-PAGE of a membrane fraction of catfish barbel (and thereby the likely localization of the putative taste receptor for L-Arg), immunohistochemistry was performed using the GP1 and GP2 antibodies pretreated as described above.

Barbels were removed from euthanized albino channel catfish (*I. punctatus*) (the fish being 5 – 7 cm in length) and immediately placed in 4% buffered paraformaldehyde (PFA) (0.1 M sodium phosphate buffer, pH 7.2–7.4) for eight hours at 4°C. After washing out the PFA with several rinses of excess buffer, the barbels were placed successively in 10%, 20%, and 30% sucrose (in buffer) for 24 hr, all at 4°C. After the final cryoprotect sucrose step, barbels were cut into pieces of less than 1 cm and mounted with M-1 Embedding Matrix (Thermo Shandon, Pittsburgh, PA). The tissue was sectioned at 10 microns on a Microm HM500OM cryostat.

For lectin histochemistry, ten micron sections of fixed barbel were incubated in the dark with rhodamine-conjugated RCA-I lectin (Vector Labs., Burlingame, CA), diluted 1/200 for 2 to 3 hr at ambient temperature. The sections were then washed quickly with Dulbecco's PBS (GIBCO/Invitrogen Corp), followed by three incubation washes of 10 min each.

For immunohistochemistry, 10 micron barbel sections, pre-washed 3 times for 10 min each in Dulbecco's PBS, were first incubated at ambient temperature for 3 to 5 hr in blocking buffer consisting of 3% bovine serum albumin, 2% goat serum, 0.3% TritonX100, and 0.1% sodium azide in Dulbecco's PBS at pH 7.1. The sections were then incubated with primary antibody, GP1 or GP2, in blocking buffer for 18 hr at 4°C. The primary antibody solution was removed and the sections were then washed once quickly with Dulbecco's PBS, followed by three incubation washes with PBS of 10 min each. The sections were then incubated in the dark with second antibody, Cy3-conjugated goat anti-guinea pig IgG (Jackson ImmunoResearch Labs., West Grove, PA) at 1:1000 dilution for 60 min. at ambient temperature. The secondary antibody was removed and the sections washed once quickly with Dulbecco's PBS, followed by three incubation washes with PBS of 10 min each. Excess fluid was removed from the slides and the sections mounted under cover slips with VectaShield (Vector Labs). Sections were observed with a Nikon Microphot FXA fluorescence microscope, photographed, and images sized and enhanced using the GNU Image Manipulation Program software [61].

Immuno-specificity was verified by running negative controls where the primary antibody was omitted from the procedure. In all cases, this control step showed no taste bud labelling (Fig. 5E). The scattered, spotty background labelling seen with the primary antibodies is likely due to both an unknown factor in pre-immune serum and to labelling of solitary chemoreceptor cells in the barbel epithelium, as was previously documented [35].

### Lipid bilayer reconstitution

Reconstitution of protein fractions containing likely L-Arg-stimulated channel activity was carried out with material from the low osmolar and galactose-eluted fractions of the lectin affinity procedure, from the protein-containing fractions off the Sephacryl S-300 gel filtration column and from each fraction of the ion exchange column. Lipid vesicles were prepared by sonication of 5 mg of DOPE:DOPC (2:1) and 0.5 ml of 5 mM Tris-HCl (pH = 7.2), 300 mM NaCl and 500 mM sucrose, to which 10 μg n-octylglucoside was added. To prepare the liposome-detergent mixture for incorporation into a lipid bilayer, approximately 0.2 to 0.5 μg of presumed receptor protein was added to the liposome in a cassette dialysis unit, and the mixture dialyzed overnight against 2000 ml of the Tris/NaCl/sucrose buffer at 4°C.

Virtually solvent-free lipid bilayer membranes were prepared as described [62]. The membrane-forming solution was an equimolar mixture of DOPS:DOPE in hexane. The bilayer chamber consisted of two symmetrical halves of a Teflon chamber, each with solution volumes of 1 ml divided by a 15 μm thick Teflon partition containing a round aperture of about 150 μm diameter. Hexadecane in n-hexane (1:10, v/v) was used for aperture pre-treatment. A pair of Ag-AgCl electrodes was connected to the solution in the chamber via 3 M KCl-4% agar bridges. "Virtual ground" was maintained at the *trans *side of the bilayer.

The bilayer was bathed symmetrically with 5 mM MOPS (pH 7.2), 1 mM CaCl_2_, 100 mM NaCl (unless otherwise stated). Fifty to 100 μl of the dialyzed liposome vesicles containing presumed receptor was added to the *cis*-side of the membrane. Fusion of the vesicles was initiated mechanically by gently mixing the membrane bathing solution from the *cis*-side using a micro-pipette. L-Arg was added to the *cis *side approximately 20 min after addition of vesicles. Unless otherwise stated all other additions of reagents also were made from the *cis *side. Channel sidedness was determined by sensitivity of the bilayer to L-Arg. The orientation of the channels was such that the L-Arg sensitive side was normally in the *cis *compartment. All bilayer experiments were performed at room temperature.

The current was amplified by a Dagan 3900 integrating patch-clamp amplifier (Dagan Corp., Minneapolis, MN) in the voltage clamp mode. Single channel data were digitized at 15 kHz (Digidata 1200, Axon Instruments, Foster City, CA) and analyzed using pClamp6 (Axon Instruments) and Origin 5.1 (Microcal Software, North Hampton, MA) software on an IBM compatible computer.

The calculated success rate of incorporation of vesicular proteoliposomes into lipid bilayers was about 25%. Success rate is defined as the ratio of the number of successful incorporation attempts to the total number of incorporation attempts (an "incorporation attempt" refers here to the formation of a new bilayer and application of putative channel protein). Over the course of these studies, the number of incorporation attempts for each fraction tested as indicated above was about ten.

## Authors' contributions

WG developed the enrichment procedures and drafted the manuscript. YK developed protein stabilization procedures and performed and interpreted the bilayer studies. DB performed the lectin- and immunohistochemistry and refined many of the enrichment procedures. AS developed the antibodies and performed pilot chromatography procedures. DLK initially designed the lectin procedure and carried out preliminary work on solubilization that made subsequent procedures possible. JT performed initial bilayer studies and advised YK on a continuing basis. JGB, the corresponding author and head of the laboratory, conceived the study, developed tissue preparations, designed and coordinated all phases of the study, and finalized the manuscript. All authors read and approved the final manuscript.

## Figures and Tables

**Table 1:** Characteristics of L-Arg stimulated ion channel activity of the three enrichment steps compared with the same parameters from the native L-Arg stimulated channels as reported by Kumazawa et al.*

Sample	+Unitary Conductance pS	Concentration of L-Arg, μM	Concentration of D-Arg, μM	Cation-anion selectivity
RCA-I lectin column material	58 ± 5			----
Sephacryl S300 column material	66 ± 3	1 – 50	10 – 200	Weak cation (P_Na_/P_Cl _= 2.2)
Ion-exchange column material	73 ± 7			--
Ion Channels from Taste Tissue	40 – 60 75–100	1 – 200	10 – 200	Cation

* From Kumazawa [31] + Data on unitary conductance reported as Mean ± SD obtained from at least three experiments.
